# Dual career competency questionnaire for athletes: psychometric properties of the Brazilian version

**DOI:** 10.3389/fpsyg.2023.1196432

**Published:** 2023-05-16

**Authors:** Guilherme Alves Grubertt, Sara Márquez, Helio Serassuelo Junior

**Affiliations:** ^1^Department of Physical Education and Sport, Federal Institute of Mato Grosso do Sul, Campo Grande, Brazil; ^2^Institute of Biomedicine (IBIOMED), Universidad de León, León, Spain; ^3^Department of Sport Sciences, Londrina State University, Londrina, Brazil

**Keywords:** dual career, student-athletes, competencies, psychometrics, factor analysis, Rasch analysis, validity

## Abstract

**Introduction:**

No studies have yet attempted to quantitatively measure the competencies of Brazilian student-athletes. Consequently, there are no validated measures suitable for this purpose. The aim of this research was to examine the psychometric properties and assess content validity, factorial validity and evidence based on response processes of the Brazilian version of the Dual Career Competency Questionnaire for Athletes (DCCQ-A).

**Methods:**

The Brazilian DCCQ-A was administered to 745 student-athletes (*M*_age_ = 17.3 ± 5.4; 54% female; 8% student-athletes with disabilities).

**Results:**

Content validity coefficient analysis indicated clarity of language, theoretical pertinence, and practical relevance of the Brazilian DCCQ-A items. Confirmatory factorial analysis revealed excellent goodness-of-fit indices of the 4-factor structure model. Polytomous Rasch analysis demonstrated an acceptable adjustment of the items and good organization in the item response categories.

**Discussion:**

Considering the psychometric strength of the Brazilian DCCQ-A, this instrument can contribute to the practical and research applicability of sport psychology, providing support to those involved with student-athletes’ dual career by identifying their competencies and possible limitations.

## 1. Introduction

A sports career is characterized by transition periods, being a long-term process with recurring stages from initiation to retirement (Wylleman et al., 2004). Holistically, in the career development of athletes, there is an ongoing interaction among several domains besides sport (psychological, psychosocial, academic/vocational, financial, and legal) that can cause significant life changes (Kegelaers et al., 2022). Based on this dynamic process, the dual career represents the whole process of performance sports training concurrently with an athlete’s educational or vocational training (Ryba et al., 2015; Stambulova and Harwood, 2022). In the last two decades, research involving dual careers has been growing in the academic and public policy scenarios (Stambulova and Wylleman, 2019) due to the possibility of identifying the demands of different transitions in sports careers, as well as potential barriers to be faced by athletes (depending on the sport and the development environment).

Dual career competencies are understood as all the skills, attitudes, and knowledge that allow athletes to adjust their educational development with their sports careers (Wylleman et al., 2017). Therefore, student-athletes have different competencies at different stages of sport development (Cosh and Tully, 2014; López de Subijana et al., 2015; Stambulova et al., 2015). Since there are several transition levels, many relevant studies have demonstrated the benefits of the sport-education combination in equal measure with its limitations (Stambulova and Wylleman, 2019). The benefits are associated with positive effects on socialization, self-regulation, employability, career management, and a balanced lifestyle (Emrich et al., 2009; Aquilina, 2013). While the costs are associated with overtraining, increased risk of injury, burnout, and premature dropout from the sport or academic context (Sorkkila et al., 2020). The European Project “Gold in Education and Elite Sport” (GEES) aimed to contribute to ensuring high-quality career support services and developed the Dual Career Competency Questionnaire for Athletes (DCCQ-A) to assess the specific dual career competencies of student-athletes. This tool allows student-athletes and their dual career support providers (parents, coaches, and friends) to focus on skill development and develop goals for their dual careers.

A critical review by Stambulova and Ryba (2014) identified more than 10 theoretical frameworks worldwide that have aimed to support discussions on student-athletes’ competencies. However, the scientific and professional communities have accepted the holistic athlete career model proposed by Wylleman et al. (2013) once it demonstrates that athletes will face transitions within the sport level and at various levels of holistic development (Torregrosa et al., 2020). According to Ricci et al. (2022), Brazil is the main country in Latin America that has the largest number of publications, and consequently, researchers involved with dual career and student-athletes that address topics such as the identification of sports and educational profiles, as well as their conciliation process based on qualitative methodologies in their majority. On the other hand, there is no alignment between data from these Brazilian studies regarding public policies aimed at the dual career of student-athletes and current discussions on this topic (Costa et al., 2018). Hence, there is a need for further debate with a theoretical and empirical background involving scientific advances in the science of physical education, psychology, education, and all other areas that affect a student-athlete through consulting with experts, athletes, clubs, and family members (Rocha et al., 2021).

Considering that the demands faced by Brazilian student-athletes are distinct during different stages of development (Miranda et al., 2020), the present study might contribute to this discussion in the Brazilian context. An instrument that qualitatively measures the attitudes and skills related to dual career of Brazilian student-athletes provides relevant data to help professionals from all spheres involved in this theme in providing targeted feedback and formulating effective interventions according to the different demands. In addition, such an instrument can impulse initiatives aimed at the life skills development, career and time management in order to align expectations and plan strategies for coping with the moment difficulties. The studies conducted in Brazil so far, which have identified and quantified dual career characteristics, have not investigated the student-athletes’ competencies using specific instruments based on a holistic approach. Thus, this research aimed to examine the psychometric properties and assess content validity, factorial validity, and evidence based on response processes of the Brazilian version of DCCQ-A.

## 2. Materials and methods

This study is a methodological, quantitative, and development adaptation and validation based on content analysis and internal structure. Burns and Grove (2005) point out that methodological studies focus on developing the validity and reliability of instruments in order to measure constructs used as variables in research.

### 2.1. Participants

Participants were 745 student-athletes (*M*_age_ = 17.3 ± 5.4; 54% female; 8% student-athletes with disabilities) beneficiaries in all categories of the Olympic and Paralympic Generation Program in the state of Paraná – Brazil. The Olympic and Paralympic Generation Program is an initiative of the Paraná State Government through Paraná Sport and the General Superintendence of Sports executed with its resources, direct sponsorships, and tax incentives authorized by the federal government through the Sports Incentive Law. Considered the broadest financial support program for athletes in Brazil, the main objective of the Olympic and Paralympic Generation Program is to provide athletes and coaches from the state of Paraná who intend to develop a sports career, with financial support based on technical performance criteria and the opportunity to dedicate themselves to their sports. This program serves as a motivating agent which contributes to the development of potential and high-performance talents for the Olympic and Paralympic Games, allowing athletes and coaches to stay in the state of Paraná while having family emotional and affective support for their performance.^1^ Basically, the categories of participants are organized into levels of competition: school (36%), state (42%), national (19%), and international (3%). Participants in the national and international categories were recognized as elite (high-performance) by their respective national elite sport institution/governing body. The student-athletes played individual-sports (68%) such as athletics (*n* = 75), boxing (*n* = 2), breaking (*n* = 1), canoe slalom (*n* = 25), cycling road (*n* = 25), sport climbing (*n* = 3), fencing (*n* = 19), wheelchair fencing (*n* = 3), artistic gymnastics (*n* = 10), rhythmic gymnastics (*n* = 41), golf (*n* = 7), equestrian (*n* = 10), judo (*n* = 48), karate (*n* = 13), weightlifting (*n* = 6), wrestling (*n* = 5), marathon swimming (*n* = 1), swimming (*n* = 73), modern pentathlon (*n* = 4), rowing (*n* = 2), skateboarding (*n* = 5), badminton (*n* = 28), surfing (*n* = 4), taekwondo (*n* = 26), tennis (*n* = 25), archery (*n* = 1), shooting (*n* = 1), triathlon (*n* = 10), boccia (*n* = 1), table tennis (*n* = 30), wheelchair tennis (*n* = 2), and team-sports (32%) such as basketball (*n* = 50), wheelchair basketball (*n =* 8), artistic swimming (*n* = 4), baseball/softball (*n* = 12), football (*n* = 21), blind football (*n* = 3), handball (*n* = 44), rugby (*n* = 11), beach volleyball (*n* = 22), sitting volleyball (*n* = 3), volleyball (*n* = 58). The student-athletes with disabilities who participated in this study attend the functional classification criteria at the national and international levels, as it is also a requisite to be a member of the Olympic and Paralympic Generation Program.

### 2.2. Instrument

DCCQ-A allows student-athletes and those involved in supporting dual careers (parents, coaches, friends) to focus on skill development and develop goals for their dual career (rather than focusing only on short-term outcome goals). This instrument was designed in English and then translated by the GEES researchers in eight languages: Catalan and Spanish (Spain), Dutch (Netherlands), French (France), Italian (Italy), Polish (Poland), Slovenian (Slovenia), and Swedish (Sweden). It consists of 29 items potentially important competencies for a successful dual career on a 5-point Likert scale (“1-Unimportant” to “5-Very important”) that measure student-athlete attitudes and result in four distinct (Dual Career Management, Career Planning, Emotional Awareness, and Social Intelligence/Adaptability) but related and conceptually meaningful dual career competency factors. The initial validation of the DCCQ-A (De Brandt et al., 2018) for the European population showed satisfactory psychometric properties [χ^2^ (296) = 2699.61, RMSEA = 0.049, CFI = 0.952, TLI = 0.934 and internal consistency (α = 0.75–0.87)].

### 2.3. Procedures

Adaptation and validation process of the Brazilian DCCQ-A followed three validity evidences of the Standards for Educational and Psychological Testing (American Educational Research Association, American Psychological Association, and National Council on Measurement in Education, 2014): content validity (study 1), factorial validity (study 2), and evidence based on response processes (study 3). Furthermore, this process also followed the main recommendations of scale validation and translation based on references widely cited in the literature (Borsa et al., 2012; Boateng et al., 2018; Cid et al., 2022). The first stage of the translation was performed by two qualified translators in order to minimize the risk of linguistic and cultural biases and theoretical and practical understanding. The second stage was to compare the different translations and evaluate their semantic, idiomatic, conceptual, linguistic, and contextual discrepancies. These comparisons and analyses were performed by a review committee composed of translators who participated in this study, doctors, researchers, professors from Brazilian public universities and with specific knowledge about the construct evaluated by the instrument (one from Psychology and two from Physical Education and Sport). The third stage was the evaluation of the structure, layout, instrument instructions, and adequacy of the expressions contained in the items conducted by the expert committee. The fourth step was performed by the target population and refers to the clarity of the instructions and the adequacy of the terms present in the items. The fifth step, recognized as back translation, was considered but not implemented because it has often been questioned (Behr, 2017). Back translation process can involve several misunderstandings and is not necessarily considered a good indicator of translation quality (Epstein et al., 2015). Previous studies indicate no clear evidence that this process improves translation quality, and this step could be omitted (Sidani et al., 2010). Finally, in the last stage, the instrument was applied to a small sample (*n* = 18) of student-athletes. These participants answered an evaluation form for the Brazilian version of the DCCQ-A, which asked about the comprehensibility of the items.

The participants were previously informed about the study procedures through three phases: (1) first contact with the General Superintendence of Sports of the state of Paraná and the GOP program to provide all the necessary information for a better grasp of the research objectives; (2) from the partnership between the Study Group on Physical Activity, Psychology and Health (GEAPS/UEL) and GOP program, researchers and coordinators responsible for this project and GOP, respectively, informed the student-athletes about the objectives and steps of this study through a document presenting the research project and an invitation to participate. This process lasted 5 months (April to August 2022). (3) The instrument was emailed to 1,100 student-athletes in September 2022, with 2 months as a deadline for fulfillment. The Brazilian DCCQ-A was conducted via an online application using the Google Forms platform. The participants completed the online survey upon providing informed consent. It is worth adding that the original instrument was also administered online (De Brandt et al., 2018).The ethics committee for research involving human beings of the State University of Londrina approved this study with the number CAAE 13654719.2.0000.5231.

## 3. Statistical analyses

The analysis process had three stages executed in sequential order. First, the content validity coefficient (CVC) was calculated to evaluate the levels of clarity of language, practical relevance, and theoretical relevance. Second, confirmatory factor analysis (CFA) was conducted to evaluate the factorial validity of the questionnaire. Third, responses to the questionnaire were submitted to psychometric analysis using the polytomous Rasch analysis. Although the sample size to be used for the development of the latent construct has often been controversial (Boateng et al., 2018), the sample size of the present study was considered very good according to Comrey and Lee (1992), a high participant-to-item ratio (26:1; Nunnally, 1978).

### 3.1. Study 1 – content validity

In order to theoretically analyze the 29 items of the Brazilian version of the DCCQ-A, the CVC was calculated for each item, called the corrected content validity coefficient (CVC_c_), and for the whole instrument (CVC_t_) proposed by Hernández-Nieto (2002). Thus, the three judges who participated in the analysis used a 5-point scale to assess the levels of clarity of language, practical relevance, and theoretical relevance of the 29 items. Based on the judges’ scores, the average of each item was calculated and, using this average as basis, the CVC_c_ of each item was calculated considering the maximum value that each item was able to receive. The calculation of the error for each item was also performed to eliminate possible biases of the judges. The calculation of the CVC_t_ was performed by subtracting the average of the items’ content validity coefficients and the average of the response bias. Although there are cut-off point recommendations of CVC > 0.70 to demonstrate satisfactory levels for the characteristics of clarity of language, practical relevance, and theoretical relevance of the items in a questionnaire, we chose to adopt a cut-off point of CVC > 0.80 as proposed by Hernández-Nieto (2002). It is important to note that an additional calculation was performed for the analysis of the questionnaire conducted by small sample (*n* = 18) of student-athletes. Although the qualitative analysis is more relevant at this stage, the same CVC calculation was conducted but applied to the small sample of student-athletes (CVC = 0.90). Microsoft Excel software was used for the CVC calculations.

### 3.2. Study 2 – factorial validity

The factorial validity of the Brazilian version of the DCCQ-A was analyzed using CFA. The model fit was evaluated using several goodness-of-fit indices and criteria; chi-square (χ^2^), Root Mean Square Error of Approximation (RMSEA) and its associated 90% confidence interval (RMSEA-CI), Standardized Root Mean Residual (SRMR), Comparative Fit Index (CFI) e Tucker-Lewis Index (TLI). The criterion scores used to indicate a good fit to the proposed model were: <0.06 for RMSEA, <0.10 for RMSEA-CI (upper), >0.95 for TLI and CFI, <0.08 for SRMR (Brown, 2015). CFA was conducted using the statistical software JASP version 16.4. Composite reliability was computed according to Raykov (1997). Usually, the estimate of reliability of an instrument is calculated using Cronbach’s alpha (α). However, this coefficient has been severely criticized as an indicator of reliability for multidimensional models due to the tau-equivalence assumption, which determines that item loadings are equal - which does not apply in this study (Green and Yang, 2009; Sijtsma, 2009; McNeish, 2018). Composite reliability of the Brazilian version of DCCQ-A was performed using the composite reliability calculator proposed by Colwell (2016). Composite reliability is an alternative that aims to overcome this limitation because it is calculated as the ratio of variance due to the common factor to the total variance of the items. Composite reliability considers the importance of each item for the construct based on its factor loadings, such as McDonald’s omega (Revelle and Condon, 2018). Although it does not seem justifiable to adopt a single, fixed cut-off point for composite reliability due to its variability as a function of items and factor loadings, values >0.70 are considered adequate (Hair et al., 2009).

### 3.3. Study 3 – evidence based on response processes (Rasch analysis – rating scale)

The psychometric properties of the Brazilian version of the DCCQ-A regarding the item response model were assessed using the polytomous Rasch analysis (Rating Scale; Andrich, 1978). The Rasch analysis involves the probability calculations of a particular person giving a particular answer to a particular question. The Logits (log odd unit) scale is a representation of the respondent’s ability to answer the test items with a varying degree of difficulty. The statistics of adjustment are the criteria of the quadratic means (MNSQ) to identify the weight or value of the information (infit) and the sensitivity of the extremes (outfit). The evaluation of the infit and outfit estimates is performed by the MNSQ and z-standardized (ZSTD). Acceptable MNSQ scores typically range from 0.7 to 1.3 logits (Bond et al., 2020), but a less conservative range of 0.5–1.5 logits can also be used (Wright and Linacre, 1994). Whereas for the ZSTD values between −2 and +2 are considered acceptable (Bond and Fox, 2007).

In addition, Rasch analysis evaluates ‘differential item functioning’ (DIF) that ensures uniformity and stability of the scale across different population groups at all difficulty levels. For this study, DIF was analyzed by sex (female, *n* = 400; male, *n* = 345). DIF was evaluated using the Mantel procedure (Mantel, 1963; Linacre, 2021a). Items whose difficulty estimates showed a statistically different value (*p* ≤ 0.05) according to the sex of the participants were inspected. The DIF magnitude, also called effect size, was interpreted using the DIF contrast. DIF contrast values between |0.00| and |0.43| are considered low/negligible; values between |0.44| and |0.64| are considered moderate and values > |0.64| are considered high, indicating that the item has no stable relationship at the same level as the latent trait, when two groups are compared (Linacre, 2021a).

An important aspect of the analysis is to verify the adequacy of the item score scale. The threshold is the transition point between two response categories on a Likert polytomous scale. Therefore, a logical ordering of response categories to the same item is expected. A threshold disorder occurs in the absence of this logical ordering, signaling that the categories are not properly used (Wu and Adams, 2007). The overall accuracy can also be measured using the person separation index, which represents the ability of a set of items to “separate” or differentiate the ability of different groups of subjects. As for reliability, the internal consistency of the questionnaire under the Rasch analysis is established using the Rasch principal components analysis of residuals. It provides estimations both for person and items; the criterion employed in this investigation to evaluate their coefficient was >0.80 (Linacre, 2021b). The polytomous Rasch analysis (Rating Scale) was conducted using the software Winsteps version 4.7.1.0.

## 4. Results

Regarding study 1, the CVC for each of the characteristics obtained acceptable values: clarity of language (0.86), practical relevance (0.88), and theoretical relevance (0.83). Likewise, the CVC_t_ of the Brazilian version of the DCCQ-A presented an acceptable value according to the cutoff point adopted (0.83). Table 1 presents the 29 items of the instrument corresponding to the English and Portuguese (Brazil) languages. Table 2 shows the descriptive data of the 29 items of the Brazilian version of the DCCQ-A. The mean of the items ranged between 3.47 and 4.57, and their variances ranged between 0.46 and 0.94. Also, it is possible to observe the asymmetry and kurtosis values of the items. Regardless of the distribution of these data, the estimation method used for CFA in this study is considered the most accepted technique currently for treating categorical data (Bandalos, 2014), being relatively stable at different levels of normality deviations and sample sizes.

**Table 1:** Competency items for the English to Portuguese (Brazil) translation of the DCCQ-A.

Item	English (original)	Portuguese (Brasil)
1.	I collaborate well with support staff in study and sport	Tenho uma boa parceria com as pessoas que me apoiam tanto no estudo quanto no esporte
2.	I can resolve conflicts	Consigo resolver conflitos que surgem com outras pessoas
3.	I understand the importance of rest and recuperation	Compreendo a importância do descanso e da recuperação
4.	I focus on here and now, without being distracted	Tenho foco no aqui e agora, sem me distrair
5.	I prioritize what needs to be done	Priorizo o que precisa ser feito
6.	I am self-disciplined to manage the demands of my study and sport combination	Sou autodisciplinado para administrar as exigências da minha combinação de estudo e esporte
7.	I am prepared for the unexpected and have back up plans	Estou preparado para o inesperado e tenho planos alternativos
8.	I have knowledge about my career options in study and sport	Tenho conhecimento das minhas opções de carreira no estudo e no esporte
9.	I am patient about the progression of my sport and study career	Sou paciente com o desenvolvimento da minha carreira no esporte e no estudo
10.	I am eager to listen and learn from others and past experiences	Estou disposto a ouvir e aprender com os outros e com as experiências passadas
11.	I can regulate my emotions in different situations	Posso controlar as minhas emoções em diferentes situações
12.	I use setbacks in sport and/or study as a positive stimulus	Utilizo as dificuldades e contratempos que acontecem no esporte e/ou estudo como estímulo positivo
13.	I am flexible and change my plans if necessary	Sou flexível e, se necessário, altero os meus planos
14.	I am dedicated to succeed in both sport and study	Estou empenhado em ter sucesso tanto no esporte como no estudo
15.	I am curious to explore career plans outside elite sport	Sou curioso para explorar planos de carreira que existem além do esporte profissional
16.	I belief that study and sport can positively complement each other	Acredito que o estudo e o esporte podem complementar-se positivamente
17.	I have a vision of where I want to go in life after my dual career	Tenho uma visão de onde eu quero ir na vida (o que quero fazer da vida) após minha dupla carreira (esporte e estudo)
18.	I cope effectively with stress in sport and study	Eu enfrento de maneira eficaz o estresse no esporte e no estudo
19.	I maintain relations with important others	Mantenho relações com outras pessoas importantes fora do esporte (família, amigos)
20.	I use my time efficiently	Uso meu tempo de maneira eficiente
21.	I make my own responsible choices with regard to my study and sport career	Faço, de maneira responsável, minhas próprias escolhas quando estas se referem ao meu estudo e carreira esportiva
22.	I have a clear understanding of what it takes to succeed in sport and study	Tenho uma compreensão clara do que é preciso ser feito para ter sucesso no esporte e no estudo
23.	I maintain relations with important others	Mantenho contatos sociais com colegas no estudo e no esporte
24.	I am willing to make sacrifices and choices to succeed in sport and study	Estou disposto a fazer sacrifícios e escolhas para ter sucesso no esporte e no estudo
25.	I create individualized routines for sport and study	Crio rotinas individualizadas para o esporte e o estudo
26.	I plan conscientiously in advance	Planejo minhas atividades estudantis e esportivas com antecedência e de maneira consciente
27.	I belief in myself to overcome the challenges in sport and study	Acredito em mim mesmo para superar os desafios do esporte e do estudo
28.	I ask advice to the right people at the right time	Peço conselhos às pessoas certas no momento certo
29.	I am assertive (I am self-assured and act with confidence)	Sou assertivo (Sou seguro de mim mesmo e ajo com confiança)

**Table 2:** Descriptive statistics of the items of the Brazilian DCCQ-A.

Item	Mean	Variance	Skewness	Kurtosis
1. I collaborate well with support staff in study and sport	4.38	0.57	−1.28	1.78
2. I can resolve conflicts	3.88	0.70	−0.52	0.23
3. I understand the importance of rest and recuperation	4.49	0.59	−1.54	2.15
4. I focus on here and now, without being distracted	3.96	0.78	−0.50	−0.40
5. I prioritize what needs to be done	4.28	0.58	−0.87	0.58
6. I am self-disciplined to manage the demands of my study and sport combination	4.16	0.67	−0.75	0.09
7. I am prepared for the unexpected and have back up plans	3.66	0.87	−0.26	−0.39
8. I have knowledge about my career options in study and sport	4.15	0.73	−0.88	0.44
9. I am patient about the progression of my sport and study career	3.94	0.73	−0.54	−0.08
10. I am eager to listen and learn from others and past experiences	4.51	0.46	−1.40	1.99
11. I can regulate my emotions in different situations	3.47	0.65	−0.14	−0.04
12. I use setbacks in sport and/or study as a positive stimulus	3.73	0.88	−0.36	−0.48
13. I am flexible and change my plans if necessary	3.74	0.84	−0.45	−0.21
14. I am dedicated to succeed in both sport and study	4.52	0.47	−1.42	1.84
15. I am curious to explore career plans outside elite sport	4.09	0.89	−0.92	0.41
16. I belief that study and sport can positively complement each other	4.57	0.49	−1.84	3.87
17. I have a vision of where I want to go in life after my dual career	4.08	0.93	−0.91	0.23
18. I cope effectively with stress in sport and study	3.62	0.77	−0.22	−0.27
19. I maintain relations with important others	4.54	0.50	−1.55	1.98
20. I use my time efficiently	3.86	0.68	−0.37	−0.26
21. I make my own responsible choices with regard to my study and sport career	4.17	0.62	−0.81	0.60
22. I have a clear understanding of what it takes to succeed in sport and study	4.22	0.63	−0.87	0.47
23. I maintain relations with important others	4.37	0.61	−1.24	1.47
24. I am willing to make sacrifices and choices to succeed in sport and study	4.41	0.56	−1.10	0.85
25. I create individualized routines for sport and study	3.99	0.86	−0.75	0.25
26. I plan conscientiously in advance	3.78	0.90	−0.49	−0.26
27. I belief in myself to overcome the challenges in sport and study	4.31	0.64	−0.99	0.49
28. I ask advice to the right people at the right time	4.02	0.92	−0.79	0.10
29. I am assertive (I am self-assured and act with confidence)	3.83	0.94	−0.46	−0.42

**Figure 1:**
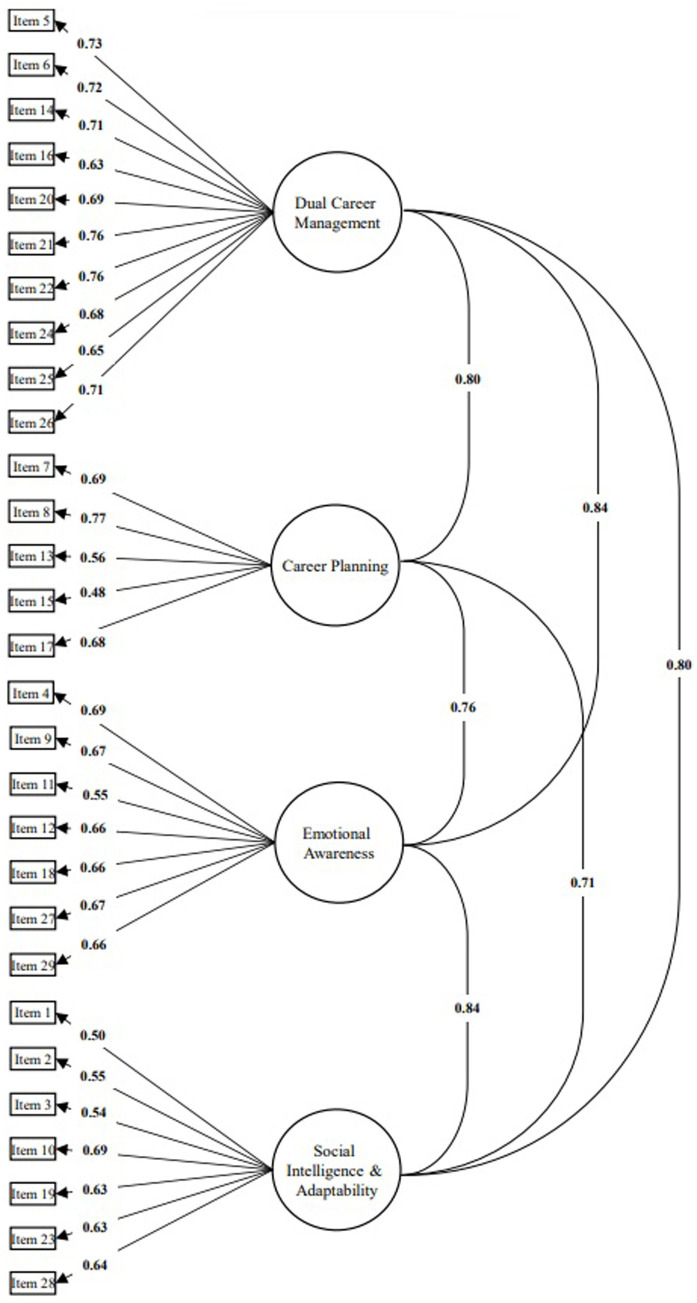
Graphical representation of the 4-factor structure model of the Brazilian DCCQ-A with its standardized primary factor loadings and inter-factor correlation coefficients.

Concerning study 2, the preliminary analysis that the factor analysis application offers is the Solomon method (Lorenzo-Seva, 2021) to divide the total sample into two equivalent samples to ensure that all possible sources of variation are included in both subsamples evidenced by the Communality Ratio Index (CRI). We verified that in the questionnaire, the two random subsamples were very similar to each other. The CRI value was close to unity (0.978), and the KMO values in each subsample of the questionnaire (Subsample 1 = 0.911; Subsample 2 = 0.931) were also close to unity and exceeded the minimum acceptable value of 0.75 (Cerny and Kaiser, 1977; Howard, 2016). Table 3 shows the goodness-of-fit indices (χ^2^, CFI, TLI, SRMR, RMSEA) of the model that proved to be acceptable except for χ^2^ because the chi-square test of exact fit is sensitive to sample size and minor model misspecifications (Marsh et al., 2005). The composite reliability and McDonald’s omega (ω) also showed acceptable values (>0.70). Figure 1 provides a graphical representation of the 4-factor structure model of the Brazilian DCCQ-A. The instrument demonstrated significant and satisfactory factor loadings ranged from 0.484 (item 15) to 0.771 (item 8). Inter-factor correlation coefficients were both high ranged from 0.712 (Career planning ↔ Social intelligence/adaptability) to 0.841 (Emotional awareness ↔ Social intelligence and adaptability). In study 3, the initial analyses showed an acceptable reliability index for the items (reliability = 0.99; separation index = 11.23) and person (reliability = 0.89; separation index = 2.84), which suggests that the estimates obtained tend to be replicable. Table 4 also shows the individual item and overall statistics such as the item difficulty estimates, infit and outfit, as well as the thresholds. It is possible to analyze that item 16 (“*I believe that study and sport can complement each other positively*”) was the easiest to endorse (δ = −1.11) and item 11 (“*I can regulate my emotions in different situations*”) the most difficult (δ = 1.16). It is important to note that the calculation of item difficulty is based on the difficulty of categories. The categories are considered the values of the Likert scale; in the case of the Brazilian DCCQ-A it is a five-point Likert scale. For example, item 11 showed the higher difficulty value in the model (Supplementary Table 1), i.e., category 1 (*n* = 6) showed an estimated latent trait mean of 0.87; category 2 (*n* = 66; δ_mean_ = 0.99); category 3 (*n* = 311; δ_mean_ = 1.54); category 4 (*n* = 297; δ_mean_ = 2.14); and category 5 (*n* = 65; δ_mean_ = 3.39). The thresholds for all items showed an ascending structure as expected theoretically. Table 5 shows the DIF values obtained for each item. Items 1, 11, 12, 18, 20, 21, 24, 25, 26, 27, and 29 obtained a value of *p* < 0.05. However, the effect sizes are considered negligible except for items 11 (DIF contrast = 0.45) and 29 (DIF contrast = 0.49) which are considered moderate values.

**Table 3:** Goodness-of-fit indices of the factorial structure model and composite reliability for the Brazilian DCCQ-A.

Model	*χ* ^2^	df	*p*	CFI	TLI	SRMR	RMSEA	[90% CI]
4-factor	1239.755	371	**	0.982	0.980	0.057	0.056	0.053–0.060

	Competencies			
DCM	CP	EA	SIA
Composite reliability	0.91	0.79	0.84	0.79
ω	0.90

*χ*^2^, chi-square fit statistic; df, degrees of freedom; CFI, comparative fit index; TLI, Tucker–Lewis Index; SRMR, standardized root mean residual; RMSEA, root mean square error of approximation; CI, confidence interval; DCM, dual career management; CP, career planning; EA, emotional awareness; SIA, social intelligence and adaptability; ω: McDonald’s omega.

** *p* < 0.001.

**Table 4:** Item difficulty, thresholds, infit and outfit estimation and global fit statistics of the Brazilian DCCQ-A.

Item	Measure	SE	Thresholds				Infit		Outfit	
			τ_1_	τ_2_	τ_3_	τ_4_	MNSQ	ZSTD	MNSQ	ZSTD
11	1.16	0.05	−0.82	0.41	1.78	3.29	0.85	−3.16	0.88	−2.50
18	0.92	0.05	−1.07	0.17	1.53	3.05	0.83	−3.62	0.83	−3.47
7	0.84	0.05	−1.14	0.09	1.46	2.97	0.96	−0.86	0.95	−0.92
12	0.73	0.05	−1.26	−0.03	1.34	2.85	0.95	−0.99	0.97	−0.60
13	0.71	0.05	−1.27	−0.04	1.33	2.84	1.11	2.14	1.18	3.28
26	0.65	0.05	−1.34	−0.10	1.26	2.78	0.91	−1.86	0.90	−2.05
29	0.55	0.05	−1.44	−0.20	1.17	2.68	1.08	1.46	1.04	0.84
20	0.50	0.05	−1.49	−0.25	1.11	2.63	0.74	−5.50	0.75	−5.21
2	0.46	0.05	−1.53	−0.29	1.08	2.59	1.00	0.05	1.03	0.54
9	0.36	0.05	−1.63	−0.39	0.97	2.48	0.87	−2.64	0.85	−2.84
4	0.32	0.05	−1.67	−0.43	0.94	2.45	0.94	−1.20	0.94	−1.09
25	0.26	0.05	−1.72	−0.49	0.88	2.39	1.07	1.32	1.09	1.62
28	0.21	0.05	−1.78	−0.54	0.83	2.34	1.22	3.97	1.18	3.10
17	0.09	0.05	−1.90	−0.66	0.71	2.22	1.30	5.22	1.28	4.68
15	0.07	0.05	−1.92	−0.68	0.69	2.20	1.50	8.27	1.54	8.38
8	−0.05	0.05	−2.04	−0.80	0.57	2.08	0.97	−0.51	0.94	−1.14
6	−0.07	0.05	−2.06	−0.82	0.55	2.06	0.85	−3.07	0.87	−2.43
21	−0.09	0.05	−2.08	−0.84	0.52	2.03	0.76	−4.88	0.75	−4.69
22	−0.21	0.05	−2.20	−0.96	0.41	1.92	0.79	−4.32	0.75	−4.67
5	−0.33	0.06	−2.31	−1.08	0.29	1.80	0.81	−3.66	0.80	−3.52
27	−0.41	0.06	−2.40	−1.17	0.20	1.71	1.03	0.63	0.98	−0.38
23	−0.54	0.06	−2.53	−1.29	0.08	1.59	1.17	2.93	1.24	3.46
1	−0.57	0.06	−2.56	−1.33	0.04	1.55	1.20	3.47	1.52	6.93
24	−0.65	0.06	−2.64	−1.40	−0.03	1.48	0.97	−0.54	0.94	−0.87
3	−0.87	0.06	−2.86	−1.62	−0.25	1.26	1.38	5.85	1.36	4.50
10	−0.94	0.06	−2.93	−1.70	−0.33	1.18	0.97	−0.46	0.90	−1.31
14	−0.98	0.06	−2.97	−1.73	−0.36	1.15	0.94	−1.04	0.90	−1.37
19	−1.04	0.06	−3.03	−1.79	−0.42	1.09	1.25	3.87	1.22	2.73
16	−1.11	0.07	−3.10	−1.86	−0.49	1.02	1.18	2.80	1.05	0.62
Global fit statistics		Separation			Reliability					
Person		2.84			0.89		1.01	−0.20	1.02	−0.10
Item		11.23			0.99		1.02	−0.10	1.02	0.10

SE = standard error; τ_1_ = threshold 1 (categories 1–2), τ_2_ = threshold 2 (categories 2–3), τ_3_ = threshold 3 (categories 3–4); threshold 4 (categories 4–5); MNSQ = mean-square; ZSTD = z standardized.

**Table 5:** Differential item functioning (DIF) sex of the Brazilian DCCQ-A.

Items	Mantel *χ*^2^	Sig.	DIF contrast
1. Tenho uma boa parceria com as pessoas que me apoiam tanto no estudo quanto no esporte	10.21	**	0.32
2. Consigo resolver conflitos que surgem com outras pessoas	0.02	0.86	0.02
3. Compreendo a importância do descanso e da recuperação	0.14	0.70	0.12
4. Tenho foco no aqui e agora, sem me distrair	0.00	0.99	0.00
5. Priorizo o que precisa ser feito	2.54	0.11	−0.15
6. Sou autodisciplinado para administrar as exigências da minha combinação de estudo e esporte	3.19	0.07	−0.18
7. Estou preparado para o inesperado e tenho planos alternativos	1.36	0.24	0.13
8. Tenho conhecimento das minhas opções de carreira no estudo e no esporte	2.25	0.13	0.16
9. Sou paciente com o desenvolvimento da minha carreira no esporte e no estudo	1.92	0.16	0.11
10. Estou disposto a ouvir e aprender com os outros e com as experiências passadas	1.16	0.27	−0.18
11. Posso controlar as minhas emoções em diferentes situações	28.52	**	**0.45**
12. Utilizo as dificuldades e contratempos que acontecem no esporte e/ou estudo como estímulo positivo	8.32	**	0.28
13. Sou flexível e, se necessário, altero os meus planos	0.33	0.56	−0.07
14. Estou empenhado em ter sucesso tanto no esporte como no estudo	1.38	0.24	−0.15
15. Sou curioso para explorar planos de carreira que existem além do esporte profissional	1.44	0.22	−0.19
16. Acredito que o estudo e o esporte podem complementar-se positivamente	3.25	0.07	−0.32
17. Tenho uma visão de onde eu quero ir na vida (o que quero fazer da vida) após minha dupla carreira (esporte e estudo)	1.57	0.20	−0.13
18. Eu enfrento de maneira eficaz o estresse no esporte e no estudo	6.54	0.01	0.21
19. Mantenho relações com outras pessoas importantes fora do esporte (família, amigos)	0.10	0.74	0.00
20. Uso meu tempo de maneira eficiente	5.33	0.02	−0.17
21. Faço, de maneira responsável, minhas próprias escolhas quando estas se referem ao meu estudo e carreira esportiva	19.19	0.00	−0.39
22. Tenho uma compreensão clara do que é preciso ser feito para ter sucesso no esporte e no estudo	2.95	0.08	−0.17
23. Mantenho contatos sociais com colegas no estudo e no esporte	0 0.12	0.72	0.00
24. Estou disposto a fazer sacrifícios e escolhas para ter sucesso no esporte e no estudo	7.37	0.00	−0.38
25. Crio rotinas individualizadas para o esporte e o estudo	5.95	0.01	−0.24
26. Planejo minhas atividades estudantis e esportivas com antecedência e de maneira consciente	15.43	**	−0.35
27. Acredito em mim mesmo para superar os desafios do esporte e do estudo	5.20	0.02	0.31
28. Peço conselhos às pessoas certas no momento certo	0.01	0.90	0.00
29. Sou assertivo (Sou seguro de mim mesmo e ajo com confiança)	19.03	**	**0.49**

Bold values are means moderate effect size; however, marginally close to the cutoff point of negligence.

** *p* < 0.01.

## 5. Discussion

Instruments in Portuguese (Brazil) that holistically measure the competencies of student-athletes in order to effectively develop them in sports were a gap in the scientific literature, so this investigation aimed to gather evidence of validity of the Brazilian DCCQ-A based on the content, internal structure, and responses processes. This instrument measures the level of importance that student-athletes consider specific dual career competencies (i.e., “*I create individualized routines for sport and study”; “I effectively cope with stress in sport and study*”).

Different statistical procedures were employed to investigate the psychometric properties of the instrument. The first result was obtained using the CVC, whose expert judges’ analysis and semantic evaluation showed satisfactory results, providing subsidies for the conclusion that the Brazilian DCCQ-A presents evidence of content validity, indicating clarity of language, theoretical pertinence, and practical relevance of the items that compose it. Although all items reached acceptable values for CVC, item 2 (“*I can resolve conflicts that arise with other people*”) and item 7 (“*I am prepared for the unexpected and have alternative plans*”) showed the lowest values for the practical relevance analysis. It is important to note that these two items compose the Social Intelligence & Adaptability and Career Planning dimensions (respectively), which also presented acceptable composite reliability values but reduced values if compared to the other dimensions. These results align with previous work by Alcaráz et al. (2020), who elaborated a method to develop short versions of instruments focused on sports psychology. In their case, the authors developed two short versions of the DCCQ-A for the Spanish context, and in both versions, the items mentioned previously were excluded.

CFA was conducted to access the second psychometric property of the instrument, which allowed a significant step in the Brazilian literature regarding student-athletes’ dual career by demonstrating excellent goodness-of-fit indices of the model composed of correlated factors. The factor structure showed to be appropriate according to the proposal of the original instrument (De Brandt et al., 2018), i.e., 29 items and four distinct but related dual career competency factors. All component items of each factor showed adequate primary factor loadings (λ ≥ 0.48). According to Howard (2016), for a factor loading to be considered acceptable, satisfactory variables should carry in their primary factor values >0.40, as occurred in the present study. Regarding inter-factor correlation coefficients, the theory of the instrument based on a holistic approach to the athletic career model itself (Wylleman, 2019) justifies this high correlation identified in the factorial structure of the Brazilian DCCQ-A. The results illustrated naturally existing interlinked relationships between the dual career competency factors because they show a consistent level of interpretability and conceptual clarity between the items.

The polytomous Rasch analysis was the third psychometric property obtained in this investigation. Diagnostic information concerning the difficulty in endorsing item 11 (“*I can regulate my emotions in different situations*”) aligns with recent discussions about the challenges and demands faced by student-athletes that influence a higher risk of mental health-related problems (Kegelaers et al., 2022) since item 11 composes a factor associated with control and how a student-athlete deals with internal and external pressures and adversity in general (Emotional Awareness). This aspect of item 11 of the Brazilian DCCQ-A corroborates the gap found in international studies since there is a need to intensify research on dual career athletes’ mental health (Stambulova and Wylleman, 2019). For example, Moesch et al. (2018) indicted practical and research recommendations addressing athletes, coaches, scholars, clubs, federations, and organizations that range from education and help-seeking behavior (a key point to normalize, prevent, and/or detect mental health problems in athletes) to developing and enhancing treatment protocols for athletes suffering from specific sport-related problems such as overtraining, psychological reactions to injury, and career termination is another important psychological reactions to injury. The pattern of responses to this item indicates the need for further research examining dual career competencies and their impact on mental health, especially in specific career transitions such as the transition from high school to college (Stambulova and Wylleman, 2019), which represents the majority of the sample in this investigation and a significant developmental stage marked by physical, psychological, and social changes. Regarding adjustment indicators (infit and outfit), it is important to note that the MNSQ tends to fit most of the items when the sample size is large, whereas the ZSTD tends to reject most of the items when the sample size is large (Wu and Adams, 2007). It is possible to observe this tendency in several items of the Brazilian DCCQ-A; however, the values found for the global indicators of item and person are within the model’s adjustment parameters, indicating that the response pattern is appropriate for this scale.

DIF analysis could check the construct equivalence across groups (Linacre, 2021a). The existence of DIF indicates that different groups may have different interpretation or perspectives on the items and, therefore, it is impossible to derive comparable measures over groups. In this study, DIF analysis was used to investigate the extent to which male and female student-athletes have performed differently on the same items. It is important to note that, like the MNSQ and ZSTD, the value of p from the DIF analysis is also sensitive to sample size. Thus, 11 of the 29 items in the instrument showed sex DIF. However, they showed a negligible effect size. On the other hand, two items (*11 – “I can regulate my emotions in different situations” and 29 – “I am assertive - I am self-assured and act with confidence*) showed a moderate effect size; however, marginally close to the cutoff point of negligence. One more time, item 11 is highlighted in the DIF analysis, supporting the importance previously reported related to aspects of the Emotional Awareness factor. Nevertheless, substantial sex DIF (moderate to high effect size) negatively impacts the measurement of the latent trait and biases the comparison between groups. Therefore, considering that Brazil is a large country, it is important that future investigations perform inferential analyses, with group tests, with larger samples, and from different regions, in order to improve the estimates of this construct. Overall, the results showed that the items of the Brazilian DCCQ-A demonstrated good fit indices for the polytomous Rasch model. It is important to note that the separation and reliability indices for person and items are replicability estimates, and the values observed in this investigation were considered appropriate. Thus, this kind of analysis has excellent practical importance. Decision-making based on the responses processes of student-athletes’ and the scores of this instrument that examines dual-career competencies requires high measurement reliability. This is the first study assessing dual career competencies of student-athletes’ in a Brazilian context with a specific instrument. No previous research on dual career of Brazilian students-athletes has recruited a sample size with the magnitude in this present investigation, including athletes with disabilities from different team and individual sports. In general, the 4-factor structure model of the Brazilian DCCQ-A that was supported by the analyses conducted in this study allows the instrument to provide an overview of sport development based on a holistic approach (Wylleman et al., 2013), as it represents different characteristics and levels of dual career development of Brazilian student-athletes.

Considering the psychometric strength of the Brazilian DCCQ-A demonstrated in this study; there are some limitations that should be mentioned. First, the sample was composed of student-athletes from the state of Paraná (located in the south of Brazil and considered the second most developed in the country). Regarding the territorial extension and the specific characteristics of each Brazilian zone, we encourage other researchers to apply the instrument with student-athletes from different country zones. Second, only a modest parcel of the sample can be considered high-performance because of their competition category (19% national level, and 3% international level), according to the Olympic and Paralympic Generation project. We consider these data a temporary limitation based on the state of the art of student-athletes’ dual career in Brazil since most studies involving this research area are associated with high-performance or talented athletes and do not involve non-Olympic sport or performance arts. Thus, we recommend starting work with the dual career competencies of Brazilian student-athletes from high school (high-performance or not; Olympic sport or not, and performance arts) seeking a better integral development during subsequent periods for this temporary limitation to be comprehended. Third, as in any self-report measure, there are always concerns regarding memory recall, bias for social desirability, and truthfulness of responses (Brenner and Delamater, 2014). In addition, considering the moment of data collection and the fact that no dual career assistance program exists, the lack of a balance between the sport and educational contexts might have originated tension when the participants were completing the questionnaire. Fourth, considering that the present study showed three categories of validity evidence and the fact that a single study does not prove validity, we encourage future investigations to include more validity evidence (validity based on relations with external variables; criterion validity, convergent, discriminant, consequential validity) and mixed research models (quantitative and qualitative approaches). Providing instruments that offer these types of validity evidence is extremely important to professionals and researchers since such evidence endorses the assumption that the variability in the instrument’s scores represents true variability in the target construct evidencing the instrument’s potential to assess this construct.

## 6. Conclusion

The present investigation reported satisfactory content validity, factorial validity, and evidence-based response process of the Brazilian DCCQ-A. It is expected that this instrument will serve as a useful tool that can help not only coaches and student-athletes to understand and optimize their attitudes and skills regarding dual career, but also researchers to continue to advance knowledge in this recent area in Brazil. Additionally, it is necessary to develop dual career research in Brazil in order to reach out to all governmental spheres because there are still no support programs for student-athletes based on this holistic approach.

## Data availability statement

The raw data supporting the conclusions of this article will be made available by the authors, without undue reservation.

## Ethics statement

The studies involving human participants were reviewed and approved by Ethics Committee for Research Involving Human Beings of the State University of Londrina. Written informed consent to participate in this study was provided by the participants’ legal guardian/next of kin.

## Author contributions

GG was involved in designing the study, collecting the data, analyzing the data, and wrote the manuscript. SM and HS reviewed and critiqued the manuscript. All authors contributed to the article and approved the submitted version.

## Funding

GG was supported by a TalentUnileon scholarship from the University of León (Spain).

## Conflict of interest

The authors declare that the research was conducted in the absence of any commercial or financial relationships that could be construed as a potential conflict of interest.

## Publisher’s note

All claims expressed in this article are solely those of the authors and do not necessarily represent those of their affiliated organizations, or those of the publisher, the editors and the reviewers. Any product that may be evaluated in this article, or claim that may be made by its manufacturer, is not guaranteed or endorsed by the publisher.

## Footnotes

^1^https://www.esporte.pr.gov.br/Pagina/Geracao-Olimpica
