# Supplementary material for: Dual career competency questionnaire for athletes: psychometric properties of the Brazilian version
Source: Front Psychol. 2023 May 16;14:1196432. doi: 10.3389/fpsyg.2023.1196432 (PMC10229064; doi:10.3389/fpsyg.2023.1196432)
Supplement: Supplementary file 1 [file Table_1.DOCX]

**Supplementary Table 1.** Item category ability of the Brazilian DCCQ-A.

| Item | Score value | N | Ability | | | MNSQ | |
| --- | --- | --- | --- | --- | --- | --- | --- |
|  |  |  | Mean | SE | | Infit | Outfit |
| 11 | 1 | 6 | 0.87  0.99  1.54  2.14  3.39 | 0.29  0.10  0.04  0.05  0.21 | | 1.3 | 1.3 |
|  | 2 | 66 |  |  |  | 1.0 | 1.0 |
|  | 3 | 311 |  |  |  | 1.2 | 1.2 |
|  | 4 | 297 |  |  |  | 1.1 | 1.2 |
|  | 5 | 65 |  |  |  | 1.1 | 1.1 |
| 18 | 1 | 7 | 0.72  0.86  1.39  2.05  3.17 | 0.20  0.10  0.04  0.04  0.13 | | 1.2 | 1.2 |
|  | 2 | 61 |  |  |  | 0.9 | 0.9 |
|  | 3 | 262 |  |  |  | 0.9 | 0.9 |
|  | 4 | 294 |  |  |  | 0.9 | 1.0 |
|  | 5 | 121 |  |  |  | 1.0 | 0.9 |
| 7 | 1 | 9 | 0.71  0.88  1.38  2.05  2.93 | 0.25  0.11  0.05  0.05  0.11 | | 1.3 | 1.3 |
|  | 2 | 64 |  |  |  | 1.0 | 1.0 |
|  | 3 | 247 |  |  |  | 0.9 | 0.9 |
|  | 4 | 273 |  |  |  | 0.9 | 0.9 |
|  | 5 | 152 |  |  |  | 1.1 | 1.0 |
| 12 | 1 | 6 | 0.90  0.79  1.35  1.98  2.89 | 0.25  0.09  0.05  0.05  0.10 | | 1.4 | 1.5 |
|  | 2 | 69 |  |  |  | 0.9 | 0.9 |
|  | 3 | 210 |  |  |  | 0.9 | 1.0 |
|  | 4 | 292 |  |  |  | 0.9 | 1.0 |
|  | 5 | 168 |  |  |  | 1.0 | 1.0 |
| 13 | 1 | 7 | 1.29  0.89  1.54  1.84  2.90 | 0.55  0.11  0.06  0.04  0.11 | | 2.2 | 2.6 |
|  | 2 | 67 |  |  |  | 1.1 | 1.1 |
|  | 3 | 191 |  |  |  | 1.3 | 1.4 |
|  | 4 | 327 |  |  |  | 1.2 | 1.1 |
|  | 5 | 153 |  |  |  | 1.0 | 1.0 |
| 26 | 1 | 9 | 0.28  0.75  1.30  1.95  2.90 | 0.26  0.10  0.05  0.04  0.10 | | 1.0 | 1.0 |
|  | 2 | 64 |  |  |  | 0.9 | 0.9 |
|  | 3 | 190 |  |  |  | 0.8 | 0.8 |
|  | 4 | 302 |  |  |  | 0.8 | 0.8 |
|  | 5 | 180 |  |  |  | 1.0 | 1.0 |
| 29 | 1 | 9 | 0.53  0.64  1.36  1.94  2.70 | 0.25  0.10  0.05  0.04  0.09 | | 1.2 | 1.2 |
|  | 2 | 57 |  |  |  | 0.8 | 0.8 |
|  | 3 | 201 |  |  |  | 1.0 | 1.0 |
|  | 4 | 259 |  |  |  | 0.8 | 0.8 |
|  | 5 | 219 |  |  |  | 1.1 | 1.1 |
| 20 | 1 | 2 | 0.29  0.60  1.20  1.90  2.95 | 0.13  0.12  0.05  0.04  0.10 | | 0.9 | 0.9 |
|  | 2 | 37 |  |  |  | 0.8 | 0.8 |
|  | 3 | 193 |  |  |  | 0.9 | 0.9 |
|  | 4 | 342 |  |  |  | 0.8 | 0.8 |
|  | 5 | 171 |  |  |  | 0.9 | 0.9 |
| 2 | 1 | 6 | 1.02  0.86  1.34  1.84  2.78 | 0.36  0.18  0.05  0.04  0.10 | | 1.7  1.2  1.0  1.2  1.1 | 1.8  1.3  1.0  1.2  1.1 |
|  | 2 | 31 |  |  |  |  |  |
|  | 3 | 181 |  |  |  |  |  |
|  | 4 | 353 |  |  |  |  |  |
|  | 5 | 174 |  |  |  |  |  |
|  |  |  | Ability | | | MNSQ | |
| Item | Score value | N |  |  |  |  |  |
|  |  |  | Mean | | SE | Infit | Outfit |
| 9 | 1 | 3 | 0.46  0.70  1.21  1.79  2.84 | 0.38  0.13  0.06  0.04  0.09 | | 1.2  1.0  0.9  0.9  1.0 | 1.2  1.0  0.9  0.8  0.9 |
|  | 2 | 40 |  |  |  |  |  |
|  | 3 | 159 |  |  |  |  |  |
|  | 4 | 339 |  |  |  |  |  |
|  | 5 | 204 |  |  |  |  |  |
| 4 | 1 | 2 | 1.15  0.70  1.25  1.75  2.78 | 0.58  0.11  0.06  0.04  0.08 | | 1.9  1.0  1.1  0.9  0.9 | 1.9  0.9  1.1  0.8  0.9 |
|  | 2 | 41 |  |  |  |  |  |
|  | 3 | 170 |  |  |  |  |  |
|  | 4 | 304 |  |  |  |  |  |
|  | 5 | 228 |  |  |  |  |  |
| 25 | 1 | 10 | 0.74  0.64  1.25  1.75  2.67 | 0.26  0.14  0.06  0.04  0.08 | | 1.5 | 1.6 |
|  | 2 | 37 |  |  |  | 1.0 | 1.0 |
|  | 3 | 152 |  |  |  | 1.0 | 1.0 |
|  | 4 | 297 |  |  |  | 0.9 | 1.1 |
|  | 5 | 249 |  |  |  | 1.0 | 1.0 |
| 28 | 1 | 10 | 0.80  0.67  1.33  1.69  2.60 | 0.24  0.12  0.06  0.05  0.07 | | 1.6  1.0  1.1  1.0  1.0 | 1.6  1.0  1.2  0.9  1.0 |
|  | 2 | 42 |  |  |  |  |  |
|  | 3 | 149 |  |  |  |  |  |
|  | 4 | 267 |  |  |  |  |  |
|  | 5 | 277 |  |  |  |  |  |
| 17 | 1 | 9 | 0.60  0.96  1.18  1.70  2.52 | 0.39  0.13  0.06  0.05  0.07 | | 1.5 | 1.7 |
|  | 2 | 47 |  |  |  | 1.4 | 1.5 |
|  | 3 | 124 |  |  |  | 1.0 | 1.0 |
|  | 4 | 261 |  |  |  | 0.9 | 0.9 |
|  | 5 | 304 |  |  |  | 1.0 | 1.0 |
| 15 | 1 | 10 | 0.84  1.18  1.40  1.65  2.44 | 0.41  0.13  0.08  0.05  0.07 | | 1.8  1.6  1.4  1.1  1.1 | 2.2  1.8  1.5  1.0  1.2 |
|  | 2 | 37 |  |  |  |  |  |
|  | 3 | 130 |  |  |  |  |  |
|  | 4 | 268 |  |  |  |  |  |
|  | 5 | 300 |  |  |  |  |  |
| 8 | 1 | 4 | -0.06  0.69  1.09  1.64  2.61 | 0.22  0.13  0.07  0.04  0.07 | | 0.9  1.1  0.9  0.9  0.9 | 0.9  1.1  0.9  0.9  0.9 |
|  | 2 | 30 |  |  |  |  |  |
|  | 3 | 113 |  |  |  |  |  |
|  | 4 | 303 |  |  |  |  |  |
|  | 5 | 295 |  |  |  |  |  |
| 6 | 1 | 2 | 0.07  0.22  1.13  1.62  2.63 | 0.50  0.16  0.07  0.04  0.07 | | 1.0  0.7  1.0  0.8  0.9 | 1.0  0.7  1.0  0.7  0.9 |
|  | 2 | 23 |  |  |  |  |  |
|  | 3 | 123 |  |  |  |  |  |
|  | 4 | 304 |  |  |  |  |  |
|  | 5 | 293 |  |  |  |  |  |
| 21 | 1 | 3 | 0.31  0.31  0.92  1.66  2.66 | 0.40  0.16  0.07  0.04  0.07 | | 1.2  0.8  0.7  0.8  0.9 | 1.2  0.8  0.7  0.7  0.9 |
|  | 2 | 20 |  |  |  |  |  |
|  | 3 | 106 |  |  |  |  |  |
|  | 4 | 335 |  |  |  |  |  |
|  | 5 | 281 |  |  |  |  |  |
| 22 | 1 | 2 | 0.55  0.11  0.93  1.60  2.61 | 0.98  0.15  0.06  0.04  0.06 | | 1.5  0.7  0.8  0.7  0.9 | 1.6  0.6  0.7  0.7  0.9 |
|  | 2 | 20 |  |  |  |  |  |
|  | 3 | 100 |  |  |  |  |  |
|  | 4 | 311 |  |  |  |  |  |
|  | 5 | 312 |  |  |  |  |  |
| Item | Score value | N | Ability | | | MNSQ | |
|  |  |  | Mean | | SE | Infit | Outfit |
| 5 | 1 | 2 | 0.07  0.10  0.91  1.59  2.53 | 0.50  0.21  0.07  0.04  0.06 | | 1.1  0.7  0.8  0.8  0.9 | 1.0  0.7  0.8  0.8  0.9 |
|  | 2 | 12 |  |  |  |  |  |
|  | 3 | 93 |  |  |  |  |  |
|  | 4 | 310 |  |  |  |  |  |
|  | 5 | 328 |  |  |  |  |  |
| 27 | 1 | 2  15  100  259  369 | 1.11  0.53  1.00  1.52  2.45 | 0.11  0.21  0.07  0.05  0.06 | | 2.1  1.1  0.9  0.9  0.9 | 2.0  1.1  0.9  0.9  0.9 |
|  | 2 |  |  |  |  |  |  |
|  | 3 |  |  |  |  |  |  |
|  | 4 |  |  |  |  |  |  |
|  | 5 |  |  |  |  |  |  |
| 23 | 1 | 3 | 0.99  0.69  1.13  1.49  2.36 | 1.01  0.18  0.11  0.05  0.06 | | 2.2  1.3  1.2  0.8  1.0 | 3.0  1.3  1.3  1.0  1.0 |
|  | 2 | 19 |  |  |  |  |  |
|  | 3 | 71 |  |  |  |  |  |
|  | 4 | 262 |  |  |  |  |  |
|  | 5 | 390 |  |  |  |  |  |
| 1 | 1 | 2 | 0.84  0.95  1.35  1.51  2.31 | 0.27  0.29  0.14  0.05  0.06 | | 1.8  1.7  1.5  1.0  1.0 | 1.8  2.3  2.4  1.1  1.1 |
|  | 2 | 20 |  |  |  |  |  |
|  | 3 | 52 |  |  |  |  |  |
|  | 4 | 291 |  |  |  |  |  |
|  | 5 | 380 |  |  |  |  |  |
| 24 | 1 | 2 | 0.20  0.48  0.88  1.46  2.38 | 0.63  0.45  0.09  0.04  0.06 | | 1.3  1.1  0.9  0.8  0.9 | 1.2  1.5  1.0  0.7  0.9 |
|  | 2 | 7 |  |  |  |  |  |
|  | 3 | 85 |  |  |  |  |  |
|  | 4 | 242 |  |  |  |  |  |
|  | 5 | 409 |  |  |  |  |  |
| 3 | 1 | 2 | 1.21  1.03  1.00  1.45  2.23 | 0.64  0.18  0.13  0.05  0.05 | | 2.5  1.8  1.2  0.9  1.1 | 2.6  1.9  1.4  0.9  1.1 |
|  | 2 | 18 |  |  |  |  |  |
|  | 3 | 60 |  |  |  |  |  |
|  | 4 | 199 |  |  |  |  |  |
|  | 5 | 466 |  |  |  |  |  |
| 10 | 1 | 1 | 0.57  0.19  0.80  1.40  2.30 | 0.01  0.29  0.12  0.05  0.05 | | 1.6  1.0  1.0  0.9  1.0 | 1.5  1.0  1.0  0.8  1.0 |
|  | 2 | 9 |  |  |  |  |  |
|  | 3 | 47 |  |  |  |  |  |
|  | 4 | 237 |  |  |  |  |  |
|  | 5 | 451 |  |  |  |  |  |
| 14 | 1 | 1 | 1.28  0.16  0.68  1.34  2.32 | 0.01  0.22  0.11  0.04  0.05 | | 2.7  0.9  0.8  0.8  0.9 | 2.5  0.9  0.9  0.8  0.9 |
|  | 2 | 8 |  |  |  |  |  |
|  | 3 | 54 |  |  |  |  |  |
|  | 4 | 218 |  |  |  |  |  |
|  | 5 | 464 |  |  |  |  |  |
| 19 | 1 | 1 | 1.23  0.99  0.89  1.40  2.22 | 0.01  0.22  0.10  0.06  0.05 | | 2.6  1.8  1.1  1.0  1.0 | 2.4  1.8  1.1  1.1  1.0 |
|  | 2 | 10 |  |  |  |  |  |
|  | 3 | 62 |  |  |  |  |  |
|  | 4 | 181 |  |  |  |  |  |
|  | 5 | 491 |  |  |  |  |  |
| 16 | 1 | 3 | 1.29  0.42  0.74  1.33  2.24 | 0.19  0.26  0.13  0.05  0.05 | | 2.8  1.2  0.9  0.8  1.0 | 2.7  1.2  0.9  0.8  1.0 |
|  | 2 | 11 |  |  |  |  |  |
|  | 3 | 42 |  |  |  |  |  |
|  | 4 | 194 |  |  |  |  |  |
|  | 5 | 495 |  |  |  |  |  |
